# Abscisic Acid: A Potential Secreted Effector Synthesized by Phytophagous Insects for Host-Plant Manipulation

**DOI:** 10.3390/insects14060489

**Published:** 2023-05-24

**Authors:** Stephannie Seng, Gabriela E. Ponce, Peter Andreas, Anna Kisiala, Rosemarie De Clerck-Floate, Donald G. Miller, Ming-Shun Chen, Peter W. Price, John F. Tooker, R. J. Neil Emery, Edward F. Connor

**Affiliations:** 1 Department of Biology, San Francisco State University, San Francisco, CA 94132, USA; stephannieseng@gmail.com; 2 Department of Entomology, The Pennsylvania State University, University Park, PA 16802, USA; gabrielaponce@gmail.com (G.E.P.); tooker@psu.edu (J.F.T.); 3 Department of Biology, Trent University, Peterborough, ON K9J 7B8, Canada; pandreas@trentu.ca (P.A.); annakisiala@trentu.ca (A.K.); nemery@trentu.ca (R.J.N.E.); 4 Agriculture and Agri-Food Canada, Lethbridge, AB T1J 4B1, Canada; rosemarie.declerck-floate@agr.gc.ca; 5 Department of Biological Sciences, California State University, Chico, CA 95929, USA; dgmiller@csuchico.edu; 6 USDA-ARS and Department of Entomology, Kansas State University, Manhattan, KS 66506, USA; ming-shun.chen@usda.gov; 7 Department of Ecology and Evolutionary Biology, Northern Arizona University, Flagstaff, AZ 86001, USA; peter.price@nau.edu

**Keywords:** abscisic acid (ABA), gall-inducing, non-gall-inducing, phytophagous, mobilizing sinks, suppression of host defenses, manipulation of host plants

## Abstract

**Simple Summary:**

Abscisic acid (ABA) is a phytohormone involved in numerous plant processes, including growth, development, and response to stress. ABA had previously been reported in a variety of animals, including insects and humans. We used high performance liquid chromatography-electrospray ionization tandem mass spectrometry (HPLC-(ESI)-MS/MS) to examine concentrations of ABA in 17 species of phytophagous insects, including gall- and non-gall-inducing species from six orders of insects. We found ABA in species in all six orders, with no tendency for gall-inducing species to have higher concentrations. The concentrations of ABA in insects often exceeded those typically found in plants, suggesting it is improbable that insects obtain all their ABA from their host plant via consumption and sequestration. We used immunohistochemistry to determine that ABA is found only in the salivary glands of the larvae of the gall-inducing *Eurosta solidaginis* (Diptera: Tephritidae). The high concentrations of ABA, combined with its presence in salivary glands, suggest that insects are synthesizing and secreting ABA to manipulate their host plants. The pervasiveness of ABA among insects and our current knowledge of the role of ABA in plant processes suggest that insects are using ABA to manipulate source-sink mechanisms of nutrient allocation or to suppress host-plant defenses.

**Abstract:**

Abscisic acid (ABA) is an isoprenoid-derived plant signaling molecule involved in a wide variety of plant processes, including facets of growth and development as well as responses to abiotic and biotic stress. ABA had previously been reported in a wide variety of animals, including insects and humans. We used high-performance liquid chromatography-electrospray ionization tandem mass spectrometry (HPLC-(ESI)-MS/MS) to examine concentrations of ABA in 17 species of phytophagous insects, including gall- and non-gall-inducing species from all insect orders with species known to induce plant galls: Thysanoptera, Hemiptera, Lepidoptera, Coleoptera, Diptera, and Hymenoptera. We found ABA in insect species in all six orders, in both gall-inducing and non-gall-inducing species, with no tendency for gall-inducing insects to have higher concentrations. The concentrations of ABA in insects often markedly exceeded those typically found in plants, suggesting it is highly improbable that insects obtain all their ABA from their host plant via consumption and sequestration. As a follow-up, we used immunohistochemistry to determine that ABA localizes to the salivary glands in the larvae of the gall-inducing *Eurosta solidaginis* (Diptera: Tephritidae). The high concentrations of ABA, combined with its localization to salivary glands, suggest that insects are synthesizing and secreting ABA to manipulate their host plants. The pervasiveness of ABA among both gall- and non-gall-inducing insects and our current knowledge of the role of ABA in plant processes suggest that insects are using ABA to manipulate source-sink mechanisms of nutrient allocation or to suppress host-plant defenses. ABA joins the triumvirate of phytohormones, along with cytokinins (CKs) and indole-3-acetic acid (IAA), that are abundant, widespread, and localized to glandular organs in insects and used to manipulate host plants.

## 1. Introduction

Abscisic acid (ABA) is an isoprenoid-derived plant signaling molecule that is involved in a wide variety of processes, including facets of plant growth and development as well as plant responses to abiotic and biotic stress [1,2,3]. For example, ABA helps regulate seed dormancy, germination, and floral induction. It plays a central role in regulating stomatal closure, thereby maintaining water balance in the face of drought, salt stress, and cold. Abscisic acid also mediates the effects of pathogen attack [4,5].

Biosynthesis of ABA in plants appears to follow an “indirect” route, which is a multistep process initiated in plastids whereby the 40-carbon carotenoid, *β*-carotene, is synthesized from the 5-carbon isoprenoid compound, isopentenyl pyrophosphate (IPP). It is further modified to form zeaxanthin and then violaxanthin before the 15-carbon molecule, xanthoxin, is cleaved from violaxanthin into the cytosol. The final two enzymatic steps lead to the synthesis of ABA via abscisic aldehyde as an intermediate [2,3,6]. A second “direct” pathway for ABA synthesis has recently been described from the fungus *Botrytis cinerea* [7,8,9]. In the direct pathway, the 15-carbon farnesyl diphosphate (FPP) is converted to α-ionylideneethane and subsequently oxidized to ABA. On the other hand, no pathway for ABA biosynthesis has yet been proposed for insects or other animals.

Abscisic acid is not uniquely of plant origin, as it has also been reported from a variety of organisms, including bacteria, fungi, sponges, hydroids, insects, and mammals (including humans), but appears to be absent from the Archaea [4,5,10,11,12,13,14,15,16,17,18,19,20,21,22]. In animals, ABA imparts a variety of physiological effects and activates a signaling pathway similar to that found in plants [11,12,15]. In human and murine systems, ABA is an activator of monocytes and smooth muscle cells, improves the survival of megakaryocytes [19,21], stimulates insulin release [23], and effects glucose uptake in skeletal muscle and adipose tissue [22], among other influences.

Abscisic acid, like cytokinins (CKs), can be viewed as an inter-kingdom signaling molecule that is involved in crosstalk with plants [24]. As such, it has been implicated as a pathogen effector capable of modulating plant defenses [4,5,25,26]. Furthermore, controlled diet experiments with rats suggest ABA’s origin in mammals is not via consumption and sequestration but rather via endogenous biosynthesis [10]. In other taxa, the source of ABA is unknown.

Two other phytohormones, CKs and indole-3-acetic acid (IAA), have often been found in gall-inducing insects [14,16,18,27,28,29,30,31,32,33,34,35]. However, recent evidence shows that both CKs and IAA are widespread and abundant in insects, including non-gall-inducing and non-phytophagous species [24,36].

Our study parallels those of [24,37] and was motivated by earlier reports of ABA in insects [14,16,18,20]. We used high-performance liquid chromatography-electrospray ionization tandem mass spectrometry (HPLC-(ESI)-MS/MS) to examine the concentrations of ABA in a wide variety of phytophagous insects from all six orders that contain species known to induce galls: Thysanoptera, Hemiptera, Lepidoptera, Coleoptera, Diptera, and Hymenoptera. We selected species that are known to induce plant galls and closely related insect species from the same order that do not induce galls. Our goal was to determine whether high concentrations of ABA were associated with gall induction or if occurrences and concentrations of ABA in gall-inducing species were similar to those of non-gall-inducing species. Furthermore, we wanted to determine if ABA was found in salivary glands, where it might be strategically produced as a secreted effector to manipulate host plants. Thus, we used immunohistochemistry (IHC) to determine if ABA, like CK and IAA, is localized in the salivary glands of the gall-inducing *Eurosta solidaginis* (Diptera: Tephritidae).

## 2. Materials and Methods

### 2.1. Insect Species

We collected 17 species of insects, including gall-inducing and closely related non-gall-inducing species, from six orders of Insecta for ABA and CK profiling. The results of the CK profiling are reported elsewhere [24]. We examined the gall-inducing stage, or, among non-gall-inducing species, the analogous stage. For a few species, we were also able to examine other life stages (Table 1 and Supplemental Table S1). The early instar larva is often the gall-inducing stage, and it can be very small (<0.2 mg). Therefore, substantial numbers of individuals were required to obtain a sufficient mass of tissue for phytohormone analyses to achieve replication (at least three biological replicates). For some insect species, ABA levels for both the juvenile and adult female stages were obtained, depending on their life cycle and interaction with the host plant.

We selected insect species based on their abundance, preferring species that were easily collected in sufficient numbers for ABA analyses so that pairs of gall- and non-gall-inducing species were as closely related as possible. Most of the sampled species’ pairs were from the same taxonomic family; however, in the Hemiptera, a congeneric pair of gall- and non-gall-inducing species was selected (*Tamalia* sp.). The insects were either wild-collected or reared, stored at −80 °C, and shipped on dry ice for chemical analysis.

### 2.2. Abscisic Acid Analysis by High Performance Liquid Chromatography-Electrospray Ionization Tandem Mass Spectrometry (HPLC-(ESI)-MS/MS)

Chemical analysis was conducted for ABA in insects. Abscisic acid was extracted and quantified using methods slightly modified from those described in [38]. No procedure was practicably possible to clear the insects’ guts before analysis. Insect samples were spiked with 144.7 ng of deuterated internal standard (^2^H_4_-ABA; NRC-PBI, Saskatchewan, SK, Canada) and homogenized in 1.0 mL of −20 °C modified Bieleski #2 extraction solvent (methanol: water: formic acid (15:4:1, *v*/*v*/*v*)) using sterile zirconium oxide grinding beads (Comeau Technique Ltd., Vaudreuil-Dorion, QC, Canada) and a Retsch 400 mixer-mill (Retsch, Haan, Germany) at 25 Hz for 5 min. Samples were passively extracted at −20 °C for 12 h, centrifuged for 10 min at 15,232 g (Thermo Scientific; Sorvall ST16, Ottawa, ON, Canada), and the supernatants collected. The remaining pellet was re-extracted using 1 mL of modified Bieleski #2 solvent at −20 °C for 1 h, and the two supernatants were combined. Supernatants were dried in a speed vac concentrator at 35 °C (Savant SPD111V, UVS400, Thermo Fisher Scientific, Waltham, MA, USA). Residues were reconstituted in 0.2 mL of 1 M formic acid (pH 1.4). Extracts were purified and fractionated on a mixed-mode, reverse-phase, cation-exchange cartridge (Waters; Oasis MCX 2cc; 96-well plate, Mississauga, ON, Canada) using an automated liquid handler system (Gilson Liquid Handler, Model 215 SPE System, Middleton, WI, USA). Cartridges were activated using 1 mL of HPLC grade methanol, followed by equilibration using 1 mL of 1 M formic acid (pH 1.4). Reconstituted samples were loaded into the MCX cartridges after equilibration. Bound residues were washed with 1 mL of 1 M formic acid (pH 1.4), and ABA was eluted with 1 mL of methanol.

ABA was analyzed by HPLC-(ESI)-MS/MS on a QTrap 5500 triple quadrupole mass spectrometer (Sciex Applied Biosystems, Framingham, MA, USA) connected to an Agilent 1100 series HPLC (Agilent, Santa Clara, CA, USA). Detection limits are given in [38]. The level of ABA is reported as the average of 3–6 replicates (±standard error) in picomoles per gram of fresh insect tissue (pmol/g fwt). Analyses were performed on whole insects or groups of insects; therefore, the reported ABA levels reflect concentrations averaged across all body tissues.

### 2.3. Collection of Eurosta Solidaginis Galls for Immunolocalization of ABA

Galls containing *Eurosta solidaginis* (Fitch) (Diptera: Tephritidae) were collected in Northfield, Minnesota, USA (N 44.467, W −93.149) on 8 and 9 July 2021 from *Solidago altissima* (Asteraceae). At that time, mostly 1st-instar larvae but also some 2nd-instar larvae were available, and galls were actively growing [27,28,39]. Galls were stored temporarily at 4 °C until the larvae were removed from the galled plant tissue. After removal from the gall, larvae were immediately put aside in sterile microcentrifuge tubes for dissection with full gut contents.

### 2.4. Insect Tissue Collection and Storage

*Eurosta solidaginis* larvae were dissected for their primary body parts at the first instar stage. Musculature (including the integument), gut (including Malpighian tubules), and salivary glands were harvested and placed directly into sterile phosphate buffered saline (PBS). The separated tissue types were stored in micro-centrifuge tubes with small amounts of PBS for fixation later the same day.

### 2.5. Fixation

Tissues were fixed under sterile conditions using 3% paraformaldehyde (PFA) in PBS for 30 min. A second fixation step was performed with fresh fixative solution on ice for 2.5 h. Samples were washed three times in PBS for 45 min for each wash and stored in PBS at 4 °C until antibody staining.

### 2.6. Immunohistochemical Staining of E. solidaginis Tissues

The dissected tissues were placed in 4% agarose (SeaPrep^®^, Lonza Rockland, Inc., Rockland, ME, USA) to reduce tissue loss during washes [40]. Tissues were stained with sterile procedures using one primary antibody (Agrisera^®^ anti-Abscisic acid [Agrisera: Cat. No. AS09 446], Vännäs, Vasterbottens Lan, SE) in blocking buffer composed of 1% bovine serum albumin (BSA) in PBS at 4 °C while rotating for 48 h in the dark. The antibody and subsequent wash reagents penetrated the agarose, where tissues remained in place to avoid losses when discarding the supernatant with a pipette. The ABA antibody recognizes ABA and ABA conjugated to the glucose ester and is known to cross-react with two precursors of ABA, abscisic aldehyde and abscisic alcohol, neither of which are usually found in plant tissues (Agrisera: Cat. No. AS09 446, product data sheet). After washing, the antibody-reacted tissues were subsequently stained with a secondary goat anti-rabbit polyclonal antibody conjugated with FITC (Vector Labs: Cat. No. BA-1000, Newark, CA, USA), which was incubated for 18 h at 4 °C, rotating in the dark.

### 2.7. Co-Localization with 4′, 6-Diamidino-2-phenylindole (DAPI)

After washing, DAPI nuclear stain (4’, 6-diamidino-2-phenylindole) was added to antibody-stained tissues to visualize the cell nuclei of the host and any chromosomes of the bacterial symbiont of *E. solidaginis*, the *Wolbachia* strain *w*Esol [41]. DAPI was added to the same tube in which tissues were stained with antibodies and incubated for 8 min. The agarose blocks with embedded tissues were placed on glass slides and melted. Tissues were mounted onto a glass slide using VECTASHIELD^®^ (Vector Labs: Cat. No. H-1000-10, Newark, CA, USA) and sealed with nail polish. After preparation, slides were placed in slide boxes at −20 °C for long-term storage in the dark. The full protocol for fixation and staining is provided in Supplemental File S1.

### 2.8. Controls

We examined various controls to rule out autofluorescence and non-specific staining. To check for autofluorescence from the insect tissues or reactivity of the reagents, we performed a negative control with buffer, washes, and no antibodies with the three isolated insect tissues individually: salivary glands, gut, and musculature. To check for cross-reactivity or non-specific binding of the primary antibody, we performed a negative control following the protocol steps, including washes and buffers, with only the primary antibody for each tissue type. To check for cross reactivity of the secondary antibody and non-specific binding by the secondary antibody, we performed a negative control following the protocol steps, including washes and buffers, with only the secondary antibody for each tissue type.

### 2.9. Visualization/Imaging

Finished slides were visualized using a Zeiss LSM 710 confocal microscope (Oberkochen, Baden-Württemberg, DE). Images were assessed to localize ABA within the sampled tissues. To visualize the co-localization of DAPI and FITC stains at the cellular level, images were acquired by taking confocal sections of each tissue sample with an EC Plan-Neofluar 40×/1.3 oil lens. All co-localized images were taken while the lasers were set for DAPI (405 nm laser, 410–497 nm emission) and FITC green (488 nm laser, 493–634 nm emission). Images were processed using Zen Blue, and images are shown with the fluorescent label used for tagging FITC depicted in green and DAPI depicted in blue.

## 3. Results

**Figure 1:**
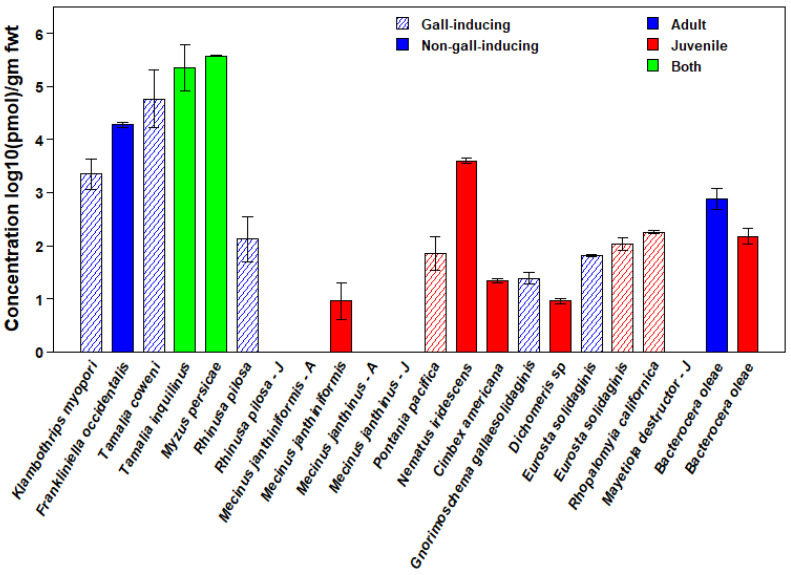
Concentrations of abscisic acid (ABA) in 17 species of gall-inducing (cross-hatched bars) and closely related non-gall-inducing species (solid bars) from six orders of Insecta. Depending on the availability of tissue samples, for some insect species, levels for both juvenile and adult stages (green), only the juvenile stage (red), or only the adult (blue) stage are shown. For species in which ABA was not detected, stage, as juvenile or adult, is indicated by a “J” or an “A” after the species name, respectively. Values are means expressed as log_10_(pmol)/g fwt. The logarithmic scale underemphasizes the large differences in concentrations among taxa. See Table 1 for means and standard errors on a linear scale.

Whole-body chemical analyses of 17 species of phytophagous insects revealed that ABA was widespread (Table 1, Figure 1). Due to the wide variation among species in ABA concentration, we plotted mean ABA concentrations on a log_10_ scale (Figure 1). However, this scale under-emphasizes the observed differences among taxa in ABA concentration (see Table 1 for means and standard errors on a linear scale). We detected ABA in at least one life-history stage in 15 species distributed across all six orders of Insecta sampled and among both gall-inducing and non-gall-inducing species. There were only two species for which ABA was not detectable at any stage, namely, ABA was not detected in either larvae or adults of the non-gall-inducing *Mecinus janthinus* Germar (Coleoptera: Curculionidae), which contrasts with the congeneric, non-gall-inducing *Mecinus janthiniformis* Toševski and Caldara, which contained ABA in larvae. We also did not detect ABA in larvae of the gall-inducing *Mayetiola destructor* (Say) (Diptera: Cecidomyiidae); however, we detected ABA in larvae of the confamilial gall-inducing species, *Rhopalomyia californica* Felt.

Concentrations of ABA were highest in the Hemiptera, averaging greater than 350,000 pmol/g fwt in both gall-inducing and non-gall-inducing species (Figure 1). Three other species had concentrations higher than 1000 pmol/g fwt, including both gall-inducing and non-gall-inducing members of the Thysanoptera (*Klambothrips myopori* Mound & Morris and *Frankliniella occidentalis* Pergande, respectively) and one species of non-gall-inducing Hymenoptera (*Nematus iridescens* Cresson, Hymenoptera: Tenthredinidae) (see Supplemental Table S1 for details on the insect species, life stages, host plants, and collecting localities).

**Figure 2:**
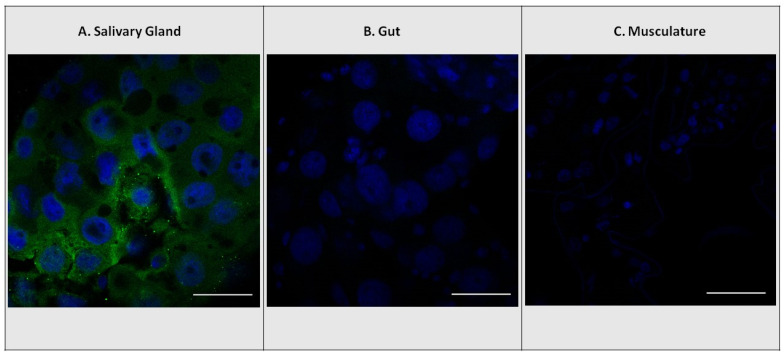
Localization of abscisic acid (ABA) antibodies with DAPI nuclear staining. Immunolocalization using rabbit ABA antibodies in *E. solidaginis* tissues with FITC-conjugated secondary goat anti-rabbit polyclonal antibody (green). Localization of ABA in (**A**) salivary gland, (**B**) gut, and (**C**) musculature tissues. DAPI stain for cell nuclei (blue). The scale bar shown in white is 50 μm.

Chemical analyses based on whole-body tissue samples of both larvae and adults revealed that *E. solidaginis* contained ABA but provided no information on the localization of ABA within specific tissues (Figure 1). However, confocal images of larval salivary glands, gut, and muscular tissues only showed antibody staining in the salivary glands (green staining), indicating that ABA was present only in the salivary glands of *E. solidaginis* (Figure 2 and Supplemental Figure S1). Control images show no evidence of autofluorescence or non-specific staining in any of the tissues (see Supplemental Figure S2 for control images).

## 4. Discussion

### 4.1. ABA Concentrations in Phytophagous Insects, Plants, and Fungi

The presence of substantial concentrations of ABA in 15 of 17 insect species suggests that ABA is widespread and abundant among phytophagous insect species. Without additional sampling, however, the lack of ABA detected in *Mecinus janthinis* and *Mayetiola destructor* should not be interpreted as evidence of its absence because ABA levels may vary diurnally, ontogenetically, or seasonally. In a separate study, ABA was detected in *Mayetiola destructor* (Antoine Guiguet, personal communication). We did not find any evidence that gall-inducing species have higher ABA concentrations than non-gall-inducing species. Our overall results suggest that insect clades differ in the amount of ABA that they tend to have. We found the highest ABA concentrations in the hemipteroid orders Hemiptera and Thysanoptera. Among the Hemiptera, all three species we examined, including the non-gall inducing *Myzus persicae* (Sulzer), *Tamalia inquilinus* Miller, and the gall-inducing *Tamilia coweni* (Cockerell), had ABA concentrations exceeding 350,000 pmol/g fwt. Two additional hemipteroid species, including both species of Thysanoptera, the gall-inducing *Klambothrips myopori* and the non-gall-inducing *Frankliniella occidentalis*, also had ABA concentrations in the thousands of pmol/g fwt. Only one other species, *Nematus iridescens*, a non-gall-inducing sawfly, contained ABA concentrations in the thousands of pmol/g fwt.

Previous studies also found ABA in insects using HPLC-MS on whole body extracts or an enzyme-linked immunoabsorbent assay (ELISA). These studies reported concentrations that span a similar range to those reported here. We supply approximate values from the literature for comparison (these values were often extracted from graphs and converted for interpretability from their native units to pmol/g fwt). Two species of psyllids have been previously examined: the gall-inducing species, *Pachypsylla celtidis* Riley, had an ABA concentration of approximately 3200 pmol/g fwt [14], whereas *Stenopsylla nigricornis* Kuwayama had ABA concentrations of 748 and 533 pmol/g fwt for nymphs feeding on leaves and flower buds, respectively [18]. In the gall-inducing *Gnorimoschema gallaesolidaginis* Riley (Lepidoptera: Gelechiidae), ABA concentrations in larvae were a function of gall diameter; however, values were highest, ~227,000 pmol/g fwt, in larvae from galls between 6–11 mm diameter [16]. Tooker and De Moraes [16] also report ABA concentrations of ~7500 pmol/g fwt in the non-gall-inducing *Heliothis virescens* (F.) (Lepidoptera: Noctuidae). These values for lepidopteran species were much higher than what we observed for *G. gallaesolidaginis* and *Dichomeris* sp., but they were similar to concentrations we reported for species in Hemiptera and Thysanoptera. Differences between our ABA concentrations and those of Tooker and De Moraes [16] for *G. gallaesolidaginis* are particularly striking. These differences might be caused by the use of different instars of caterpillars in different assays. Tooker and De Moraes [16] did find much lower ABA concentrations in young larvae. Acevedo et al. [20] directly examined concentrations of ABA in the saliva of *Spodoptera frugiperda* (J.E. Smith) (Lepidoptera: Noctuidae) using HPLC-MS and found that ABA concentrations varied from 7.5 to 20 pmol/g fwt among different insect strains and the same strain feeding on different diets. Based on ELISA and a stomatal-closure bioassay, Heath et al. [42] concluded ABA was present in salivary glands of the flat morph of the gall-inducing *Asteromyia carbonifera* (Osten Sacken) (Diptera: Cecidomyiidae) at concentrations between 0.5 and 1.5 pmol/g fwt. Wang et al. [35] also reported ABA concentrations in the gall-inducing *Leptocybe invasa* Fisher & LaSalle (Hymenoptera: Eulophidae) as high as 264 pmol/g fwt based on ELISA assays.

Concentrations of ABA in plants and fungi appear to depend on the physiological state of the plants and tissues examined, but concentrations are often substantially lower than those reported in insects. While we found no comprehensive review of ABA concentrations in plants or fungi, we report values from a variety of plant and fungal species under different environmental conditions. ABA concentrations in the epidermis and mesophyll tissues of leaves of *Tulipa gesneriana* L. (Liliaceae) of 46 and 57 pmol/g fwt were detected, respectively [43]. Similarly, for *Commelina communis* L. (Commelinaceae), ABA concentrations in epidermis and mesophyll tissues were 30 and 53 pmol/g fwt [43]. Hein et al. [44] reported ABA concentrations in developing seeds of *Glycine max* (L.) Merr. (Fabaceae) of ~3800 pmol/g fwt for cotyledons and ~64,000 pmol/g fwt along the embryonic axis. Tossi et al. [45] reported ABA concentrations in seedling leaves of *Zea mays* L. (Poaceae) of ~230 pmol/g fwt, with values doubling 4 h after exposure to UVB radiation. In *Solanum lycopersicum* L. (Solanaceae), ABA concentrations were reported in the leaves and roots of unstressed plants of 114 and 38 pmol/g fwt, respectively, and as high as 1173 and 2080 under severe salt stress treatments [46]. Negin et al. [47] report an ABA concentration in wild-type *Arabidopsis thaliana* (L.) Heynh. (Brassicaceae) of ~680 pmol/g fwt and as high as 984 pmol/g fwt in some transgenic lines. In a survey of twenty species of forest fungi, including ectomycorrhizal, wood-rot, and saprotrophic species, ABA was detected in all species with concentrations ranging between 1–25 pmol/g fwt [17].

### 4.2. Localization of ABA in the Salivary Glands of Eurosta solidaginis

Our immunohistochemical examination of the tissues of *E. solidaginis* indicated that ABA was found uniquely in the salivary glands (Figure 2). This result is similar to that reported for CK and IAA in *E. solidaginis* [37]. The localization of ABA in the salivary glands of *E. solidaginis* combined with the high concentration suggests that *E. solidaginis* is secreting ABA into its host plant at concentrations that are physiologically relevant to its interactions with its host plant.

Evidence from other insect species and for other phytohormones (e.g., CK and IAA) is consistent with the interpretation that these compounds are produced to be secreted into the host plant. Other phytophagous insect species are known to have high concentrations of phytohormones associated with glandular structures capable of delivering their contents to the host plant. Yamaguchi et al. [30] found CK and IAA localized in the accessory glands associated with the ovipositor of adult females of *Pontania* sp. (Hymenoptera: Tenthredinidae), the life stage that induces the gall. Brütting et al. [48] found that *Tupiocoris notatus* (Distant) (Hemiptera: Miridae) secreted CKs into its host plant while feeding, implying that they are associated with salivary glands as well. As mentioned above, ABA was also detected in the saliva of *S. frugiperda* [20] and in the salivary glands of *A. carbonifera* [42].

It is possible that ABA is present in other insect tissues at concentrations below detectable levels for IHC. The fact that ABA has been detected and has a physiological function in a diverse array of animal groups suggests it may also play a role in the physiology of *E. solidaginis* and in other insect species [4,10,11,12,13,15,19,21,22]. Further examination of specific tissues of insect species using HPLC-MS could verify the distribution of ABA.

### 4.3. Acquisition of ABA by Insects

The concentrations we report for ABA in insects are largely based on whole-body tissue extractions and do not necessarily reflect concentrations in glands involved in secreting effectors into the host plant. However, given the localization of ABA to the salivary glands in *E. solidaginis*, the actual concentration in the salivary glands must be substantially higher than the whole-body concentration (if the quantity of ABA was expressed as a function of salivary gland mass). Even without a more accurate picture of glandular concentrations, whole-body concentrations of ABA in most species would be unlikely to be achieved via consumption and sequestration. Similar observations on the possible synthesis of CKs in insects have also been reported [14,24,49]. Given the low concentrations of ABA in plants relative to the concentrations of these phytohormones in insects, generating the observed concentrations in insects would require them to consume a prohibitive mass of plant tissue and to possess the ability to efficiently extract and sequester ABA from their host plant.

Although relative quantities in plants and insects strongly support the possibility that insects make their own ABA, it could be argued that microorganisms associated with insects are the source of ABA. Microbial symbiosis has been proposed as the source of CKs and IAA in the induction of galls and green islands [49,50,51,52,53]. Bacteria are known to induce plant galls via manipulation of plant biosynthesis of CKs and IAA or by secretion of CKs and IAA into plants [54,55,56,57,58,59]. A green-island-inducing moth, *Phyllonorycter blancardella* (F.) (Lepidoptera: Gracillariidae), has a *Wolbachia* sp. symbiont that appears to be responsible for CKs secreted by the caterpillar while feeding [50]. However, Hammer et al. [60] examined the set of insect species we report on here using bacterial 16s ribosomal RNA gene amplicon analysis. They found no evidence of a common microbial symbiont or a community of microbial symbionts shared by gall-inducing insects but absent from non-gall-inducing species. Ponce et al. [37] also report that chromosomes of the *Wolbachia* symbiont of *E. solidaginis, w*Esol, were absent from the salivary glands where CKs, IAA, and now ABA have been detected. Finally, Fiutek et al. [41] sequenced, assembled, and annotated the genome of *w*Esol and concluded that *w*Esol lacked the metabolic pathways to supply *E. solidaginis* with either CKs or IAA. Cumulatively, these results undermine the notion of microbial symbiosis as a widespread mechanism of gall induction and of provisioning insects with CKs or IAA. Furthermore, CKs, IAA, and ABA are now known to be widespread in insects and not just in gall-inducing species ([24,36] and this study). We contend that the widespread presence of CKs, IAA, and ABA in insects does not arise from microbial symbiosis, although it could in specific instances [60].

Because consumption and sequestration and microbial symbiosis are unlikely to account for ABA in insects, biosynthesis is the likely source. Whether insects use the indirect synthesis pathway, similar to plants, the direct pathway, similar to fungi, or some as yet undescribed pathway will require further observation and experiments. The indirect pathway for ABA synthesis begins with the 40-carbon carotenoid, *β*-carotene [2,3,6]. Insects in general cannot synthesize carotenoids, so use of the indirect pathway would require that insects obtain carotenoids from their diet.

A few groups of insects, including aphids, phylloxerids, adelgids, and cecidomyiids, have acquired the ability to synthesize carotenoids via horizontal gene transfer [61,62,63]. Furthermore, the whitefly *Bemisia tabaci* (Gennadius) (Hemiptera: Aleyrodidae) has a bacterial symbiont capable of supplying carotenoids [64]. It is intriguing to speculate that the very high concentrations of ABA we report for aphids are related to their ability to synthesize carotenoids, which may serve as the starting point for ABA synthesis via the indirect pathway [2,3,6]. However, some species in these carotenoid-synthesizing groups, such as *S. nigricornis* (Psyllidae) and *M. destructor* (Cecidomyiidae), had low concentrations or no detectable ABA. Therefore, the presence of the genes for carotenoid biosynthesis may only provide the opportunity to acquire high concentrations of ABA, and the levels of expression for these genes and ultimately ABA concentrations may subsequently evolve in response to selection.

### 4.4. Role of ABA in Host-Plant Manipulation by Insects

The presence of high concentrations of ABA in a wide variety of phytophagous insects, coupled with its localization in the salivary glands of *E. solidaginis*, *Spodoptera frugiperda*, and *Asteromyia carbonifera,* suggests that phytophagous insects synthesize and secrete ABA to manipulate their host plants. However, at this point, we have no idea about the quantities or the time course of ABA secretion in even a single insect-plant interaction—not to mention the formidable task of understanding the effects of ABA on plant-herbivore interactions. However, insect manipulation remains a critical line of inquiry as ABA plays important roles in stomatal closure [65], regulating anthocyanin biosynthesis [66,67,68,69,70], strengthening mobilizing sinks [70,71,72], and modulating host-plant defenses [25,73,74,75,76,77].

There is considerable evidence that insects manipulate plant stomata, and the secretion of ABA by insects may play a pivotal role in such manipulation [65]. High ABA concentrations result in stomatal closure, which can lead to increased water retention and temperature of plant tissues, reduced emissions of volatile organic compounds (VOCs), and resistance to invasion of plant pathogens via the stomata [65]. Each of these consequences of stomatal closure could also benefit feeding insect herbivores.

Anthocyanins are common plant pigments that give rise to red and purple coloration. While not commonly and visibly associated with insect-feeding sites, they are often associated with insect-induced galls [78]. CKs are often credited with regulating anthocyanin biosynthesis; however, ABA also plays a positive role [66,67,68,69,70]. Regulation of anthocyanin synthesis appears to involve an interaction of sugar, hormones, and light, with CKs and ABA playing positive roles, whereas ethylene (ET) and gibberellins (GA) have negative regulatory roles [66]. Therefore, we suggest that not only CKs but also ABA synthesis and secretion account for the red coloration of plant galls exposed to sunlight.

Cytokinins are widespread in phytophagous insects, and their role in the formation of mobilizing sinks has been hypothesized as one of the main functions of CK biosynthesis and secretion in insects [24]. Similarly, ABA appears to be involved in the strengthening of mobilizing sinks [70,71,72], which would complement the known role of CKs in establishing and strengthening sinks [48,79,80,81].

Probably the most important effect of ABA in insect-plant interactions is the modulation of plant defenses. Abscisic acid has been shown to modulate salicylic acid (SA) and jasmonic acid/ethylene (JA/ET)-mediated plant defenses [25,73,74,75,76,77], is involved in crosstalk with other phytohormones [82,83,84], and has been reported to affect the expression of over 1000 different genes [85]. Therefore, understanding the role of exogenous insect-supplied ABA in plant defense and its impact on phytophagous insects will be devilishly difficult.

While some studies implicate ABA as a negative regulator of plant defense [25,75,76,86,87], other studies suggest it can play a positive role [88,89,90,91], and the effect of ABA on herbivores may depend on host-plant species and the feeding guild of the herbivore species [92]. Perhaps a starting point to resolve this conundrum would be to examine, both biochemically and transcriptomically, the responses of the SA and JA/ET pathways in a host plant to the feeding of an insect from a manipulable feeding guild with a known ABA concentration. In any event, much more research is necessary to fully understand how ABA affects plant defenses against herbivores.

## 5. Conclusions

ABA joins other phytohormones (e.g., CK and IAA) that are abundant, widespread, and localized to glandular organs in insects and that are capable of being delivered to the host plant. It is possible that ABA plays other physiological roles within insects, but considering its localization within the insect body, its primary role may be in host-plant manipulation. A broader survey of ABA concentrations and localization within insects would be useful to solidify the pattern we observed. Experiments to determine the physiological role of ABA in insects and its pathway of biosynthesis are also needed. Given the crosstalk among CKs, IAA, and ABA and their sometimes antagonistic interactions, further understanding of the roles of these three phytohormones in host-plant manipulation by insects will require experiments targeting concentrations and time courses of secretion of all three phytohormones and their individual and combined effects on the host plant. These details will be particularly relevant at the location of insect feeding, but also at larger scales. Finally, given the detection of yet more phytohormones in insect saliva (e.g., SA, JA [20]) and the detection of JA, GA, and brassinosteroids in a gall-inducing insect species [35], a more comprehensive examination of non-protein effectors in insect saliva is essential to produce a more complete picture of the repertoire of effectors phytophagous insects may use to manipulate their host plant species.

## Figures and Tables

**Table 1:** ABA concentrations (pmol/g fwt) in sampled species.

Species	Order	Family	Galling	Life Stage	Mean ABA Concentration	Standard Error	Number of Biological Replicates
*Frankliniella occidentalis*	Thysanoptera	Thripidae	no	adult	19,142.17	2441.86	3
*Klambothrips myopori*	Thysanoptera	Phaleothripidae	yes	adult	4174.64	1548.43	5
*Tamalia coweni*	Hemiptera	Aphididae	yes	adult female	471,959.52	212,072.50	6
*Tamalia inquilinus*	Hemiptera	Aphididae	no	nymphs & adults	466,718.05	169,034.96	4
*Myzus persicae*	Hemiptera	Aphididae	no	nymphs & adults	370,746.10	10,167.15	5
*Rhinusa pilosa*	Coleoptera	Curculionidae	yes	adult female	245.75	115.56	3
*Rhinusa pilosa*	Coleoptera	Curculionidae	yes	larvae	0.00	0.00	3
*Mecinus janthinus*	Coleoptera	Curculionidae	no	adult female	0.00	0.00	2
*Mecinus janthinis*	Coleoptera	Curculionidae	no	larvae	0.00	0.00	2
*Mecinus janthiniformis*	Coleoptera	Curculionidae	no	adult female	0.00	0.00	4
*Mecinus janthiniformis*	Coleoptera	Curculionidae	no	larvae	24.37	13.46	5
*Pontania pacifica*	Hymenoptera	Tenthredinoidea	yes	larvae	118.18	76.55	3
*Nematus iridescens*	Hymenoptera	Tenthredinoidea	no	larvae	4118.47	477.27	5
*Cimbex americana*	Hymenoptera	Tenthredinoidea	no	larvae	22.18	1.75	3
*Dichomeris* sp.	Lepidoptera	Gelechiidae	no	larvae	9.21	1.20	3
*Gnorimoschema gallaesolidaginis*	Lepidoptera	Gelechiidae	yes	larvae	27.40	6.08	5
*Bacterocera oleae*	Diptera	Tephritidae	no	adult	961.15	471.05	3
*Bacterocera oleae*	Diptera	Tephritidae	no	larvae	200.21	89.39	5
*Eurosta solidaginis*	Diptera	Tephritidae	yes	female	118.56	34.53	3
*Eurosta solidaginis*	Diptera	Tephritidae	yes	larvae	53.07	6.12	7
*Rhopalomyia californica*	Diptera	Cecidomyiidae	yes	larvae	181.41	13.42	4
*Mayetiola destructor*	Diptera	Cecidomyiidae	yes	larvae	0.00	0.00	6

## Data Availability

The data presented in this study are available on request from the corresponding author.

## Supplementary Materials

The following supporting information can be downloaded at: https://www.mdpi.com/article/10.3390/insects14060489/s1, Figure S1: Grayscale Treatment Images; Figure S2: Control Images; Table S1: Tissue sampling for HPLC-MS/MS analysis of ABA; File S1: Immunohistochemical Whole Mounted Tissue Protocol for Antibodies for Abscisic Acid.
