# Supplementary material for: Abscisic Acid: A Potential Secreted Effector Synthesized by Phytophagous Insects for Host-Plant Manipulation
Source: Insects. 2023 May 24;14(6):489. doi: 10.3390/insects14060489 (PMC10299484; doi:10.3390/insects14060489)

Controls: No antibody with DAPI staining

Green-488nm laser (493-634 nm emission) & Blue-405 nm laser (410-497nm emission)

Salivary Gland

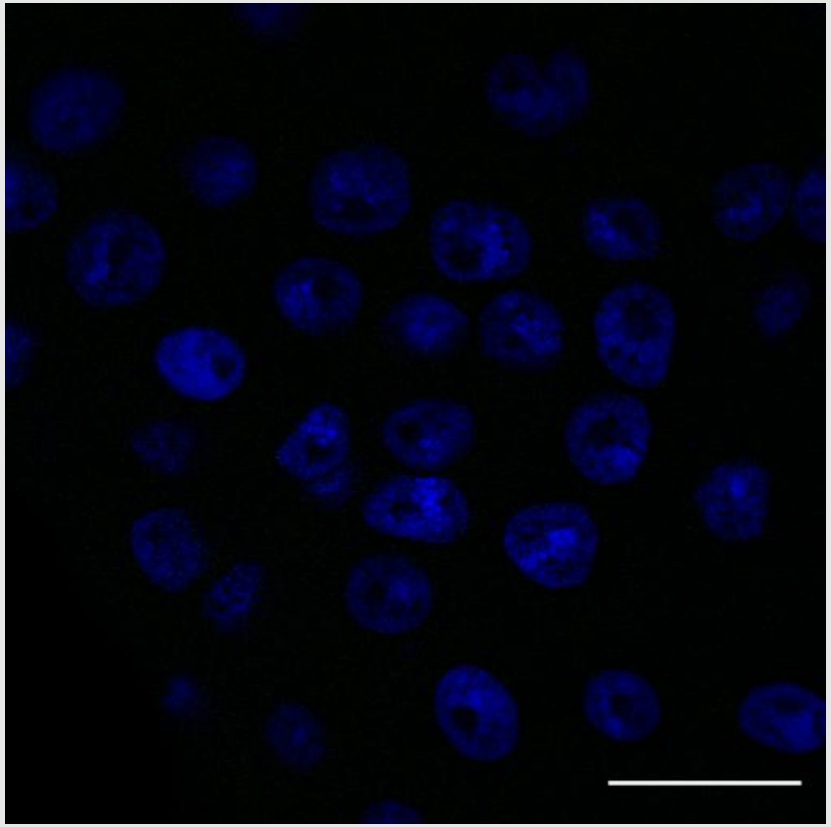

Gut

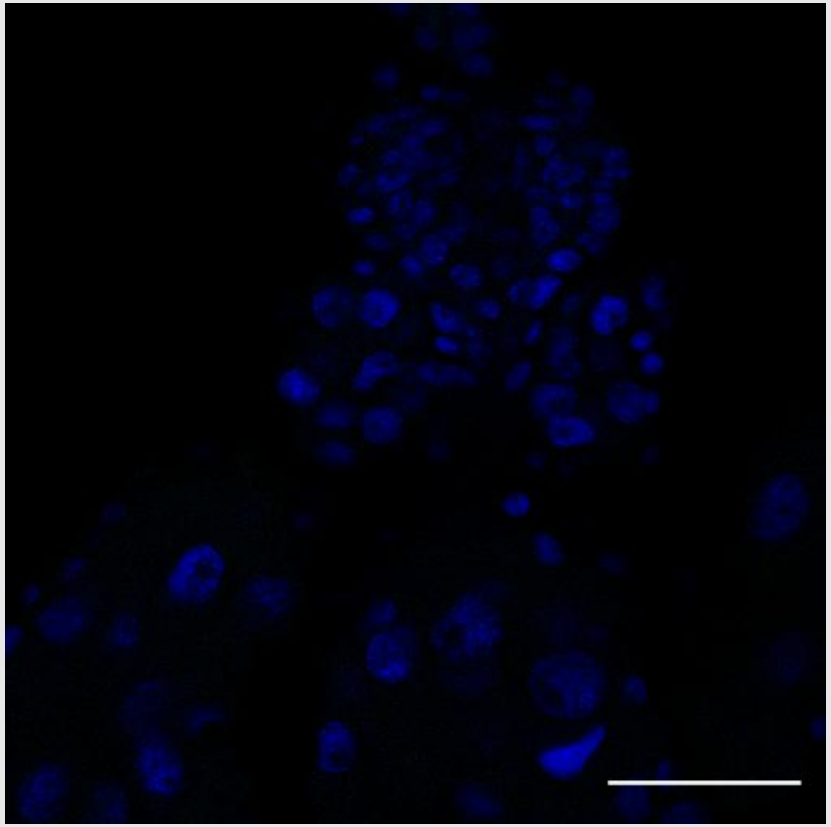

Musculature

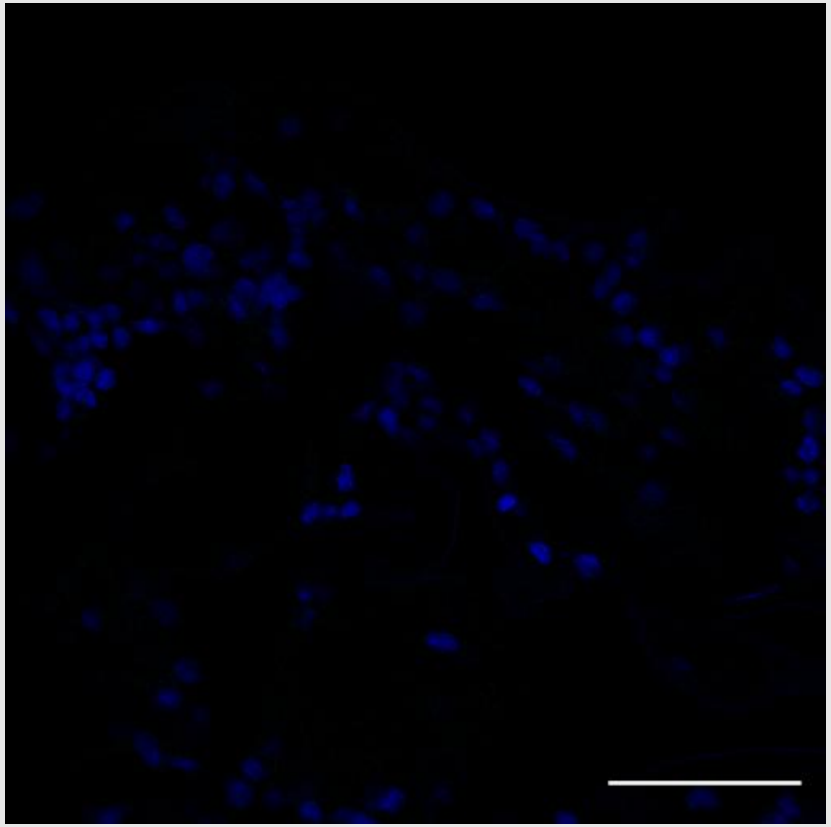

Controls: Primary antibody only with DAPI

Green-488nm laser (493-634 nm emission) & Blue-405 nm laser (410-497nm emission)

Salivary Gland

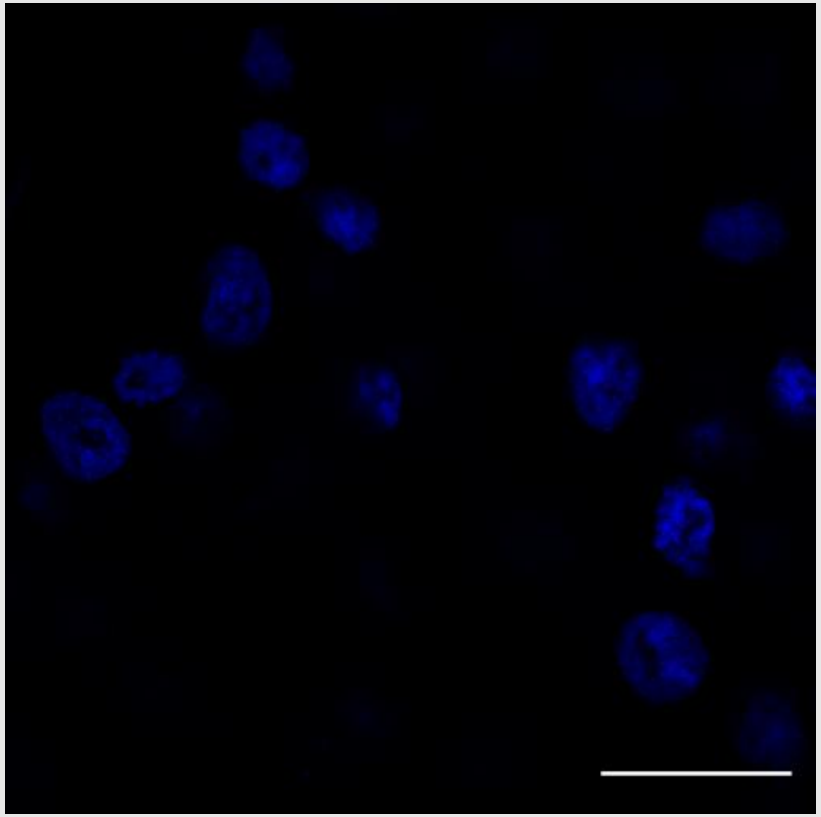

Gut

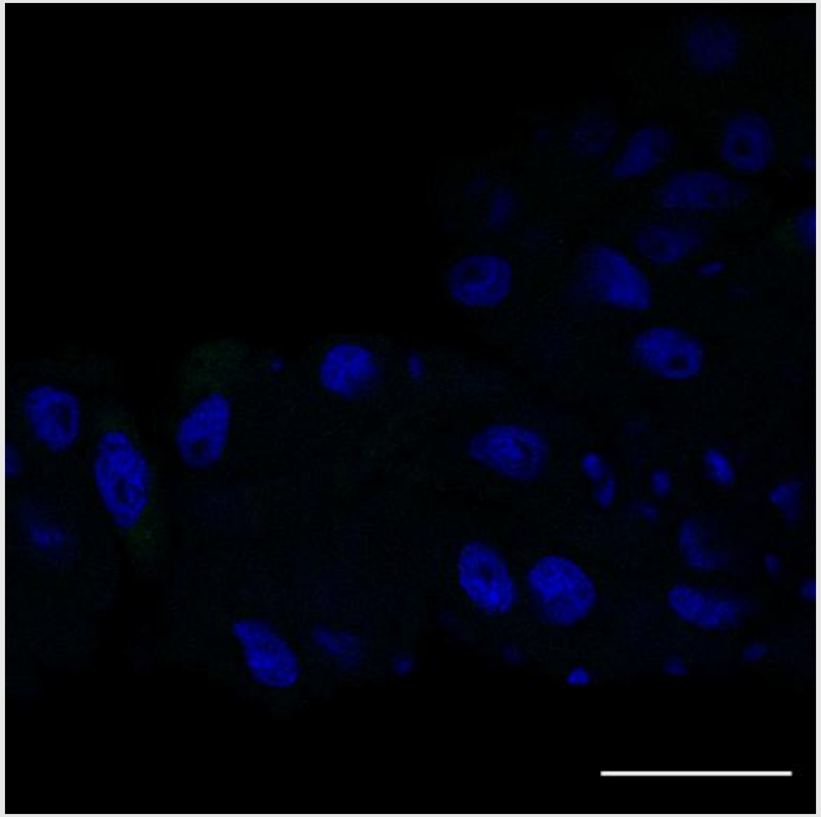

Musculature

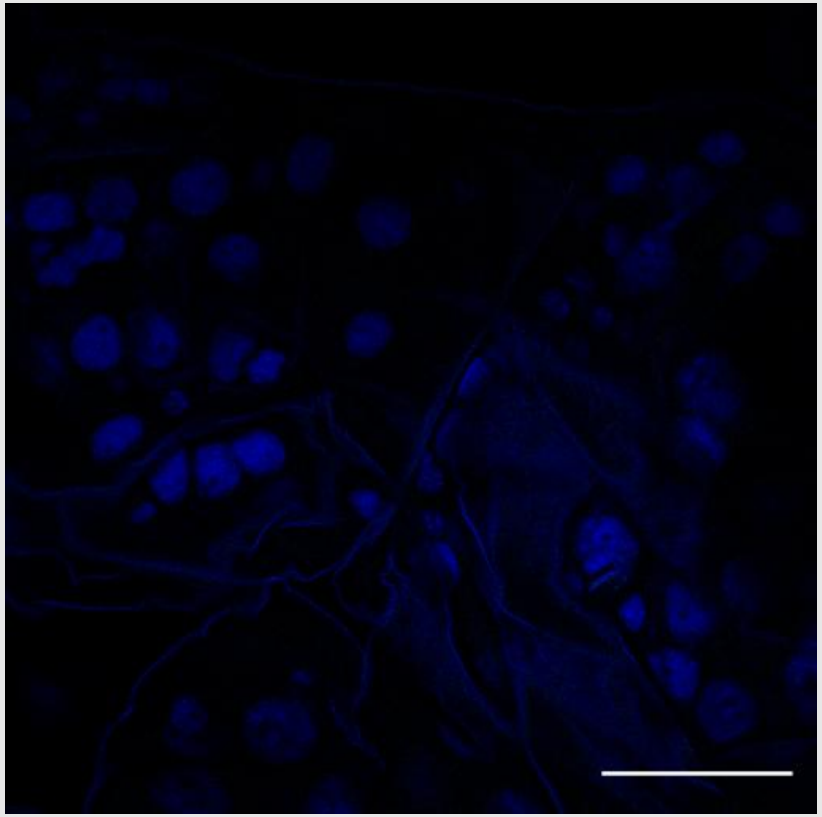

Controls: Secondary antibody only with DAPI

Green-488nm laser (493-634 nm emission) & Blue-405 nm laser (410-497nm emission)

Salivary Gland

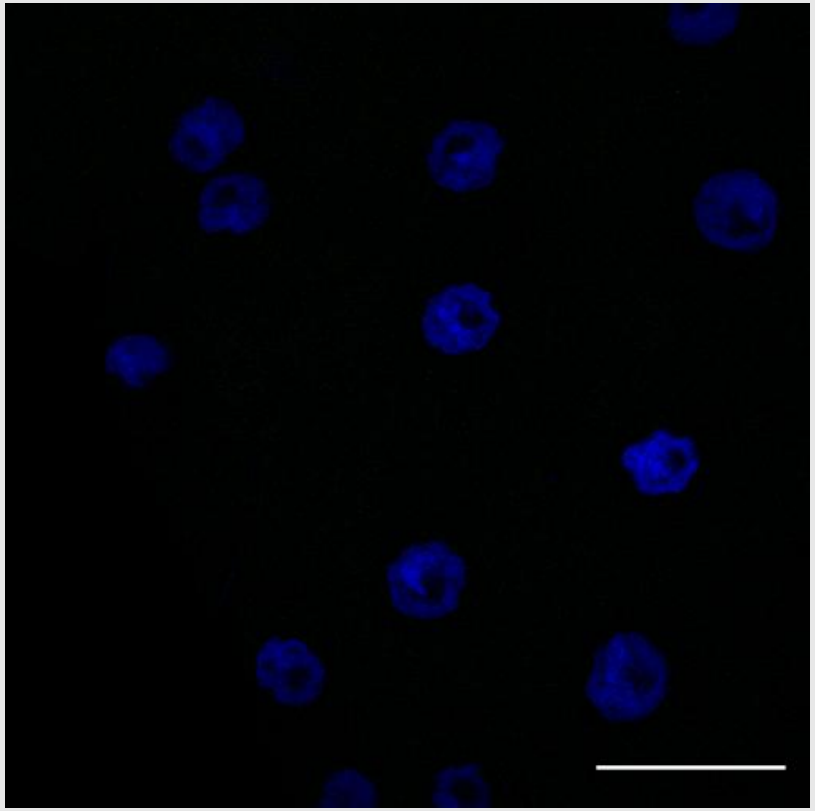

Gut

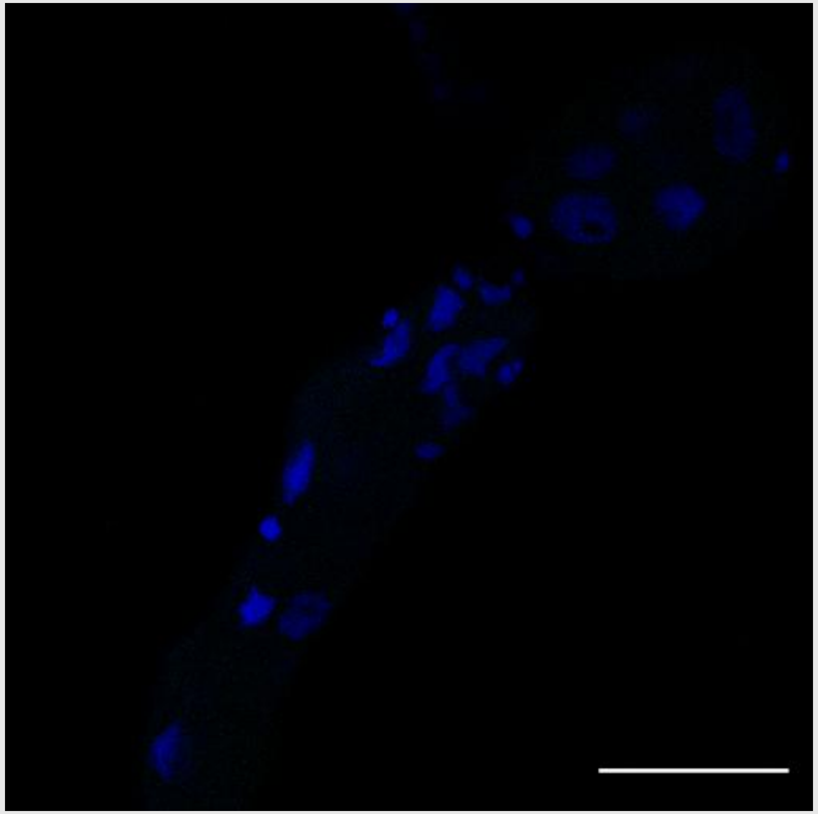

Musculature

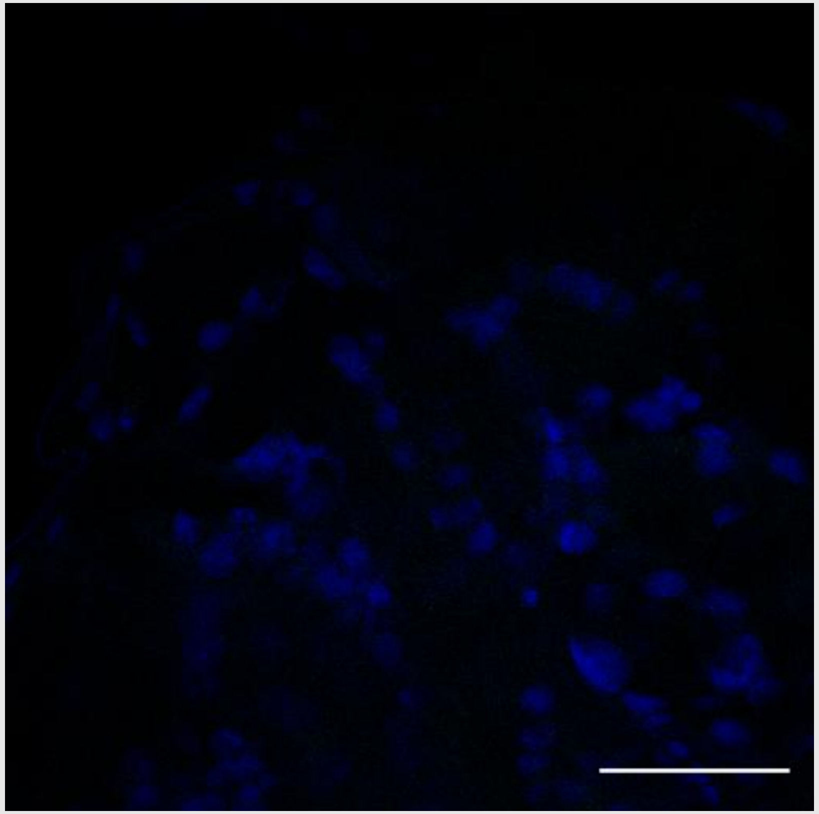

Supplement: Supplementary file 1 [file insects-14-00489-s001.zip › ABA_Supplemental Figure S2 - Controls.pdf]
