# Supplementary material for: Abscisic Acid: A Potential Secreted Effector Synthesized by Phytophagous Insects for Host-Plant Manipulation
Source: Insects. 2023 May 24;14(6):489. doi: 10.3390/insects14060489 (PMC10299484; doi:10.3390/insects14060489)
Supplement: Supplementary file 1 [file insects-14-00489-s001.zip › Supplemental File-Immunohistochemical Whole Mounted Tissue Protocol for Antibodies for Abscisic Acid.pdf]

Immunohistochemical Whole Mounted Tissue Protocol for Antibodies for Abscisic Acid co-localized with DAPI within *Eurosta solidaginis*

Dissection:

Check if animal is alive by poking and detecting movement, color should be semi-opaque/ white/light yellow. Dark yellow and no movement means sample is not viable.

1. Wash off detritus from larvae in PBS before dissection.
2. Dissect larvae by holding denticles and then slicing anterior to posterior with microblade.
3. Then remove tissues from body wall by prying it away with minuten.
4. Place in PBS while collecting other samples in 2ml centrifuge tubes filled with 25µl of sterile PBS.

Fixation:

1. Place whole mounted body part (gut, salivary glands and musculature) in cold fixative solution of 3% paraformaldehyde in PBS for 30 minutes.
2. remove initial fixative, and incubate samples in fresh fixative solution on ice for 2.5 hours.
3. Wash samples 3 times in PBS, 45 minutes for each wash and then store in PBS at 4°C overnight.

Remove Fixative:

1. Wash samples with blocking buffer PBS-BSA (1% BSA) 3 times, 10 minutes for each wash.

2. Wash samples in for 30 minutes in PBSBT (PBS/Triton 0.1%)

Anti-body Staining:

1. Prepare a 4% solution of SeaPrep Agarose®. Dissolve agarose in DNA free water on a stirring hotplate.
2. Transfer samples to 0.05 ml microcentrifuge tubes and add SeaPrep Agarose® to cover tissues.
3. Add primary antibody solution to the tubes (1:250 anti-ABA antibody in 1% BSA) and incubate in the dark for 48 hrs at 4° C with rotation.
4. Bring samples to room temperature on the bench, then wash with PBS five times, 10 minutes each wash with shaking.
5. Add the secondary antibody (goat anti-rabbit - FITC conjugated) diluted 1:1000 in PBS to the samples and incubate for 18 hours in the dark at 4C with rotation.
6. Wash 5 times for 10 min each in PBSBT with shaking.
7. Use micro blade to remove agarose plug from tube, transfer onto slide, and place slide on hotplate to melt agarose.
8. (skip this step if co-locating with DAPI) Add a drop of VECTASHIELD® as mounting agent, add cover slip, and seal with nail polish.

For co-localization + DAPI:

1. Dilute the DAPI stock solution to 1:1000 in PBS. Add approximately 300 µL of this dilute DAPI staining solution to the microcentrifuge tube.
2. Incubate for 8 minutes.

3. Wash in PBS 3 times for 10 minutes each wash with shaking.

Slide Preparation

1. Use micro blade to remove agarose plug from tube, transfer onto slide, and place slide on hotplate to melt agarose.
2. Add a drop of VECTASHIELD® as mounting agent, add cover slip, and seal with nail polish.

Reagents:

§ Phosphate Buffered Saline (PBS x1M)

§ 3% paraformaldehyde in Phosphate Buffered Saline (PBS)

§ 1% Bovine Serum Albumin

§ PBT (PBS + .1% triton)

§ PBSB (PBS + 1% BSA)

§ PBSBT (PBS + .1% triton and 1%BSA)

§ 1/250 anti-ABA in PBSB.

§ DAPI (4′,6-diamidino-2-phenylindole)

§ VECTASHIELD®

§ SeaPrep Agarose® 3%
