# Supplementary material for: Abscisic Acid: A Potential Secreted Effector Synthesized by Phytophagous Insects for Host-Plant Manipulation
Source: Insects. 2023 May 24;14(6):489. doi: 10.3390/insects14060489 (PMC10299484; doi:10.3390/insects14060489)
Supplement: Supplementary file 1 [file insects-14-00489-s001.zip › Supplemental Table S1.pdf]

Order (Family)	Gall Inducer	Non-gall Inducer	Life Stage	Host Plant	Locality	Site-Collector
**Thysanoptera**<br>(Phlaeothripidae)	*Klambothrips myopori*		Adult	*Myoporium laetum*	Pacifica, CA	Field-EFC
(Thripidae)		*Frankliniella occidentalis*	Adult	*Rosa sp.*	Half Moon Bay, CA	Greenhouse-EFC
**Hemiptera**<br>(Aphididae)	*Tamalia coweni*		Adult	*Arctostaphylos viscida*	Chico, CA	Field-DGM & EFC
		Tamalia inquilinus	Adult + Nymph	*Arctostaphylos viscida*	Chico, CA	Field-EFC
		Myzus persicae	Adult + Nymph	*Salix lasiolepis*	San Francisco, CA	Field-EFC
**Coleoptera**<br>(Curculionidae)	*Rhinusa pilosa*		Adult & Larvae	*Linaria vulgaris*	Lethbridge, CAN	Lab Colonies-RDF
		Mecinus janthinis	Adult & Larvae	*Linaria vulgaris*	Lethbridge, CAN	Lab & Field colonies-RDF
		Mecinus janthiniformis	Adult & Larvae	*Linaria dalmatica*	Fort Macleod, CAN	Field-RDF
**Hymenoptera**<br>(Tenthredinoidea)	*Pontania pacifica*		Larvae	*Salix lasiolepis*	Pacifica, CA	Field-EFC
		Cimbex americana	Larvae	*Salix lasiolepis*	Pacifica, CA	Field-EFC
		Nematus iridescens	Larvae	*Populus angustifolia X P. fremontii*	Flagstaff, AZ	Field-PWP
**Lepidoptera**<br>(Gelechiidae)	*Gnorimoschema gallaesolidaginis*		Larvae	*Solidago altissima*	University Park, PA	Field-JFT
		Dichomeris sp.	Larvae	*Solidago altissima*	University Park, PA	Field-JFT
**Diptera**<br>(Tephritidae)	*Eurosta solidaginis*		Adult & Larvae	*Solidago altissima*	Northfield, MN	Field-EFC
		Bactrocera oleae	Adult & Larvae	*Olea europea*	Santa Rosa, CA	Field-EFC
(Cecidomyiidae)	*Mayetiola destructor*		Larvae	*Triticum aestivum*	Manhattan, KS	Greenhouse-MSC
	Rhopalomyia californica		Larvae	*Baccharis pilularis*	San Francisco, CA	Field-EFC

Supplemental Table S1. Tissue sampling for HPLC-MS/MS analysis of ABA.

In cases where two life stages were collected: & indicates both stages were analyzed separately, + indicates stages were pooled. Collectors are indicated by abbreviated names: Rosemarie De Clerck-Floate (RDF), Edward F. Connor (EFC), Ming-Shun Chen (MSC), John F. Tooker (JFT), Peter W. Price (PWP), Donald G. Miller (DGM)
