# Supplementary figures and images for: Abscisic Acid: A Potential Secreted Effector Synthesized by Phytophagous Insects for Host-Plant Manipulation
Source: Insects. 2023 May 24;14(6):489. doi: 10.3390/insects14060489 (PMC10299484; doi:10.3390/insects14060489)

DAPI

FITC

Combo (DAPI+FITC)

M  
u  
s  
c  
u  
l  
a  
t  
u  
r  
e

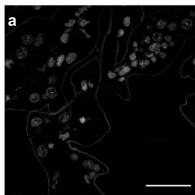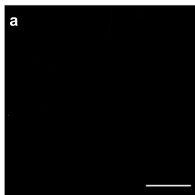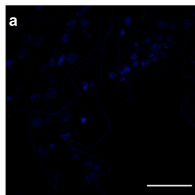

S  
a  
l  
i  
v  
a  
r  
y

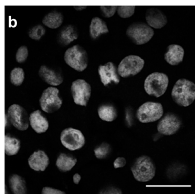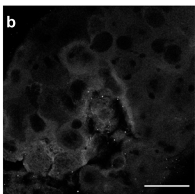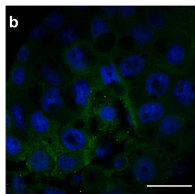

G  
u  
t

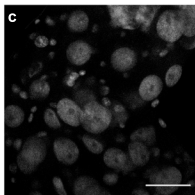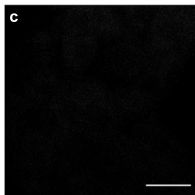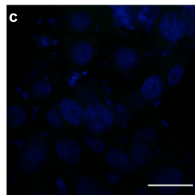

Supplement: Supplementary file 1 [file insects-14-00489-s001.zip › ABA_Supplemental Figure S1 - Grayscale Treatment Images.pdf]
